# Constancy checks of well‐type ionization chambers with external‐beam radiation units

**DOI:** 10.1120/jacmp.v16i6.5608

**Published:** 2015-11-08

**Authors:** Sara L. Hackett, Benjamin Davis, Andrew Nixon, Ruth Wyatt

**Affiliations:** ^1^ Department of Radiotherapy University Medical Center Utrecht Utrecht The Netherlands; ^2^ Medical Physics Department University Hospitals Birmingham NHS Foundation Trust Birmingham UK

**Keywords:** well‐type ionization chamber, quality assurance, constancy check

## Abstract

Constancy checks of a well‐type ionization chamber should be performed regularly as part of a quality assurance regime. The goal of this work was to test the feasibility of using a linear accelerator and an orthovoltage unit to check the constancy of a well‐type chamber's response to an external radiation source. The reproducibility, linearity with dose, variation with dose‐rate, and variation between energy‐matched units of the well‐type chamber response when exposed to 6 MV beams was examined. The robustness to errors in establishing the measurement conditions, including setting the source‐to‐surface distance and gantry angle, rotation of the chamber around the central axis of the beam, and the effect of changing the length of the chamber cable exposed to the field, were tested. The reproducibility and linearity with dose of the chamber response, and robustness to errors in establishing the measurement conditions for 100 kVp and 250 kVp beams from an orthovoltage unit, were also examined. The combined uncertainty, including contributions from errors in establishing the reference conditions, for well‐type chamber measurements using a 6 MV beam from a linear accelerator is 1.0%. The combined uncertainties for measurements using 100 and 250 kVp beams were 1.8% and 1.5%, respectively. When focus‐source distance errors were reduced to ≤1mm, the combined uncertainties for the 100 and 250 kVp beams were 1.2% and 1.1%, respectively, when the dose to the chamber was confined to the linear region of the dose‐response curve. The response of a well‐type chamber should remain constant to within 1.2% when exposed to a constant dose from an external beam unit, if reference conditions can be reproducibly established. However, the uncertainty for establishing reference conditions for output measurements for an orthovoltage unit can be reduced, which would justify a reduction of the tolerance for constancy measurements.

PACS numbers: 87.55.Qr, 87.56.Fc

## I. INTRODUCTION

Well‐type ionization chambers are used for calibration of both high‐ and low‐dose‐rate brachytherapy sources. A quality assurance program for any instrument used for dose calibration is essential, and should include a regular check of the constancy of the instrument calibration.^1^, ^2^ The International Atomic Energy Agency recommends that the constancy of a well‐type chamber calibration be checked with a ^137^Cs source or an external beam ^60^Co unit.^(3)^ However, ^60^Co teletherapy units are rarely used in developed countries, and many departments no longer use ^137^Cs sources for brachytherapy. We have developed an alternative means of checking the constancy of a well‐type chamber to external beam sources using a linear accelerator and an orthovoltage X‐ray unit. The purpose of this study was to demonstrate that external beam units can be used as part of a quality assurance system for well‐type chambers by assessing the constancy of the chamber measurements when exposed to external X‐ray beams and the reproducibility of positioning the chamber under each external beam unit.

## II. MATERIALS AND METHODS

### A. Linear accelerator measurements

The response of a well‐type ionization chamber to 6 MV beams on a pair of energy‐matched Elekta Precise linear accelerators (Elekta AB, Stockholm, Sweden) was assessed. The well‐type chamber readings were corrected for dose output of the specific linear accelerator, as measured under reference conditions with a PTW (Physikalisch‐Technische Werkstätten GmbH, Freiburg, Germany) Farmer chamber type 30013, connected to a PTW UNIDOS E electrometer, and for air temperature and pressure.

**Figure 1:**
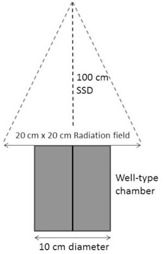
Experimental setup for well‐type chamber measurements with a linear accelerator. The solid vertical line is the chamber vertical axis, the dashed vertical line is the central axis of the radiation beam, and the angled vertical lines indicate the beam edges.

A PTW well‐type chamber, connected to a PTW UNIDOS E electrometer, was positioned on the treatment couch of a linear accelerator, as illustrated in Fig. 1. The standard conditions for the measurements were a source‐to‐surface distance (SSD) of 100.0 cm, a field size of 20 cm×20 cm, a dose rate of approximately 600 monitor units (MUs) per min, and a gantry and collimator angle of 0°, with the center of the well‐type chamber positioned at the intersection of the light‐field crosshairs. Unless stated, all measurement conditions were set to these standard conditions. No source calibration insert was used. The collection voltage on the electrometer was set to 400 V and the electrometer range set to ‘High’ (corresponding to a range of 20 nC−65 mC). The chamber and electrometer were left to stabilize for 10 min. The chamber was then exposed to five beams, each delivering 200 monitor units (MU), equivalent to approximately 1.1 Gy at the surface of the chamber. The median of the electrometer readings was considered to be the reference reading, and all results are reported as a percentage of the reference reading.

Reproducibility was calculated as the standard deviation (SD) of the five readings delivered under reference conditions. Variation with dose rate was assessed by delivering two beams of 200 MU at dose rate of 300 MU per min, and reported as the difference between the reference reading and the median of the readings recorded at lower dose rate. Linearity with dose was assessed by delivering beams of 50, 100, 200, and 400 MU. The variation of linearity with dose reported as maximum difference between the measured accumulated charge per MU and the reference reading. The uncertainty associated with establishing the standard conditions for measurements were simulated by delivering two beams of 200 MU at gantry angles from 0.1° to 0.5° at 0.1° intervals, and then with the SSD set to 100.2 cm and the gantry angle set to 0.0°. The effects of gantry angle and SSD errors were reported as the difference between the reference reading and the median of the readings obtained with the nonreference gantry angles and with the nonreference SSD, respectively. The variation between energy‐matched machines was assessed by positioning the chamber on a second linear accelerator, under standard conditions, and delivering five beams of 200 MU. The variation was reported as the difference between the median of the five readings and the reference reading.

The effects of rotation of the chamber and of exposing the co‐axial cable connecting the chamber to the electrometer were assessed using a different well‐chamber and electrometer, as the original set was no longer accessible. A Standard Imaging HDR 1000 PLUS (Standard Imaging Inc., Middleton, WI) well‐type ionization chamber, connected to a second PTW UNIDOS E electrometer, was positioned on the treatment couch of an Elekta Precise linear accelerator. A new reference reading was obtained by exposing the chamber to five beams of 200 MU under the standard conditions described above. The effect of rotating the chamber about the central axis of the beam was examined by delivering four beams of 200 MU with the chamber rotated by 90° between beams and reported as the difference between the median of these readings and the reference reading. The effect of exposing the cable was assessed by increasing the length of cable within the X‐ray field on the treatment couch from approximately 5 cm to approximately 95 cm (the full length of the well‐type chamber cable) and delivering four beams of 200 MU. The cable effect was reported as the difference between the median of these readings and the reference reading.

### B. Orthovoltage unit measurements

The response of a Standard Imaging HDR 1000 PLUS well‐type ionization chamber, connected to a PTW UNIDOS E electrometer, to 100 kVp and 250 kVp X‐rays from a Gulmay D3225 orthovoltage unit (Gulmay Medical Ltd., Surrey, UK) was assessed. All well‐type chamber readings were corrected for air temperature and pressure.

**Figure 2:**
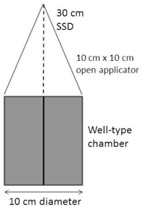
Experimental setup for well‐type chamber measurements with an orthovoltage unit, using a 100 kVp beam and an open circular applicator. The solid vertical line is the chamber vertical axis, and the dashed vertical line is the central axis of the radiation beam and applicator.

**Figure 3:**
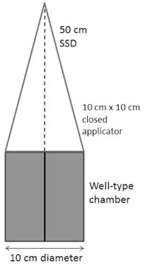
Experimental setup for well‐type chamber measurements with an orthovoltage unit, using a 250 kVp beam and a closed square applicator. The solid vertical line is the chamber vertical axis, and the dashed vertical line is the central axis of the radiation beam and applicator.

The well‐type chamber was positioned on the treatment couch of the orthovoltage unit. A 10 cm×10 cm applicator with an open end and a focus–surface distance (FSD) of 30.0 cm was used for all 100 kVp measurements, while a 10 cm×10 cm applicator with a closed end and an FSD of 50.0 cm was used for all 250 kVp measurements. The experimental setups for the 100 kVp and 250 kVp measurements are shown, respectively, in Figs. 2 and 3. The center of each applicator was positioned over the center of the chamber. For the 250 kVp measurements, the surface of the applicator was flush with the surface of the chamber and, for the 100 kVp measurements, the edges of the open‐ended applicator were flush with the chamber surface. The gantry angle was set to 0°. No source calibration insert was used. Unless stated, all measurement conditions were set to these standard conditions. The respective dose rates measured in air at 100 kVp and 250 kVp are 442 cGy per min and 88 cGy per min at the end of the applicator and cannot readily be adjusted. The collection voltage of the electrometer was set to 400 V, the electrometer range set to ‘High’, and the chamber and electrometer were left to stabilize for 10 min. The potential voltage of the orthovoltage unit was set to 250 kVp and the chamber was then exposed to five beams of 200 cGy. A further five beams of 200 cGy were delivered with the potential voltage set to 100 kVp. The medians of each set of five readings were considered the 250 kVp and 100 kVp reference readings. All results obtained at each potential voltage are reported as a percentage of the corresponding reference reading.

Reproducibility of readings, linearity with dose, and uncertainty associated with establishing the standard measurement conditions, including off‐axis positioning of the chamber, were assessed at both voltage settings. Reproducibility and linearity with dose were assessed as described for the linear accelerator measurements. The effects of errors in positioning the chamber under the X‐ray source were tested at both voltage settings by delivering two beams of 200 cGy with the gantry angle set to 0.5° and with the FSD increased by 0.2 cm. The effects of gantry angle and FSD errors were reported as the difference between the reference reading and the median of the readings obtained with the nonreference gantry angle and FSD, respectively. Note that when the gantry angle was adjusted, the end of the applicator was no longer parallel with the surface of the chamber. The effect of positioning the chamber off the central axis of the beam was assessed by shifting the chamber 0.2 cm on each cardinal axis, and delivering a beam of 200 cGy at each new position. The effect was reported as the difference between the median of the readings at each off‐axis position and the reference reading. The effect of rotation of the chamber was examined at 250 kVp, as described for the linear accelerator measurements. No assessment of the effect of increasing the length of cable included in the X‐ray field was made, as the chamber occupied most of the area of the primary field.

## III. RESULTS & DISCUSSION

The results for the linear accelerator and orthovoltage measurements are shown in Tables 1 and 2, respectively. The combined uncertainty, $u_{c}$, for well‐type chamber measurements for the external beam units was calculated using the formula in Eq. (1). The combined uncertainty comprises the uncertainty for absolute calibration of the unit, $u_{\text{abs}\_\text{cal}}$, the uncertainty associated with establishing reference conditions for the well‐type chamber measurements, $u_{\text{ref}\_\text{cond}}$, and the sources of variability of the measurements, $u_{\text{var}}$.

(1) $$u_{c}^{2}=u_{abs\_cal}^{2}+u_{ref\_cond}^{2}+u_{\text{var}}^{2}$$

(2) $$u_{abs\_cal}^{2}=u_{stability}^{2}+u_{abs\_ref\_cond}^{2}$$

(3) $$u_{ref\_cond}^{2}=u_{SSD}^{2}+u_{gantry}^{2}+u_{angle}^{2}+u_{cable}^{2}$$

(4) $$u_{\text{var}}^{2}=u_{rep}^{2}+u_{linear}^{2}+u_{dose\_rate}^{2}+u_{linac}^{2}$$

The uncertainty for absolute calibration of the external beam unit, calculated using Eq. (2), comprises the long‐term stability of the dosimeter used for beam calibration, $u_{stability}$, and the uncertainty associated with establishment of reference conditions, $u_{abs_{\_}ref\_cond}$. The uncertainty associated with establishing reference conditions for the well‐chamber measurements, shown in Eq. (3), includes the uncertainties arising from setting the *SSD* (or FSD for orthovoltage measurements), $u_{SSD}$, and the gantry angle, $u_{gantry}$, the variations introduced by the chamber rotation, $u_{angle}$, and the length of cable irradiated by the beam, $u_{cable}$. The variability of the well‐type chamber measurements, $u_{var}$, given in Eq. (4), comprises the uncertainty associated with reproducibility of the measurements, $u_{rep}$, linearity with dose, $u_{linear}$, variation with dose rate, $u_{dose\_rate}$, and variation between linear accelerators, $u_{linac}$.

The combined uncertainty for well‐type chamber measurements using a 6 MV beam from a linear accelerator is 1.0% of the reference dose measurement, which is with the same as the recommended tolerance of ± 1.0% for constancy of chamber readings using a ^60^Co unit.^(3)^ The error of 0.31% introduced by a change of gantry angle from 0.0° to 0.5° indicates the uncertainty introduced by using only the gantry angle indicator only to set the gantry angle, as the recommended tolerance for the accuracy of gantry angle indicators^(4)^ is 1.0°. If a more accurate means of setting the gantry angle is used, a gantry angle error of 0.2° would introduce only a 0.03% error for the measurements, although this would not change the combined uncertainty of 1.0% for the constancy of chamber readings.

Thus, it is reasonable to expect that chamber readings for 6 MV beams from a linear accelerator should be constant within ±1.0%, after correction for beam output and air temperature and pressure, and the required accuracy of positioning of the chamber in the beam is achievable. The small variation of 0.5% between readings obtained on machines with TPR_20,10_ values of 0.588 and 0.592 indicates that the chamber readings will not be unduly affected by the variations of beam energy within the recommended tolerance of 1.0% of TPR_20,10_ from baseline.^(4)^

The combined uncertainty for well‐type chamber measurements using 250 kVp and 100 kVp beams from an orthovoltage unit are 1.5% and 1.8%, respectively. Nonlinearity with dose constitutes the largest source of error of the 100 kVp measurements; the accumulated charge per cGy is 1.1% lower for the 50 cGy beam than the 200 cGy beams, whereas the accumulated charge per cGy for the 100 and 400 cGy beams are within 0.1% of the accumulated charge per cGy for the reference readings. The observed nonlinearity also includes nonlinearity of the output of the orthovoltage unit and/or timer errors. The results demonstrate the importance of restricting the dose used for constancy checks to the linear region of the dose‐response curve of the well‐type chamber and of the output of the orthovoltage unit.

**Table 1:** Estimated relative standard uncertainty of chamber response for linear accelerator measurements

*Physical Quality or Procedure*	*Relative Standard Uncertainty (%)*
Step 1: Absolute dose calibration of linear accelerator	
Long‐term stability of dosimeter	0.3
Establishment of reference conditions	0.4
Combined uncertainty of step 1	0.5
Step 2: Establishment of well‐chamber reference conditions	
Gantry angle uncertainty of 0.5°	0.31
SSD uncertainty of 2 mm	0.58
Angle of chamber relative to central axis of beam	0.11
Length of cable in field	0.02
Combined uncertainty of step 2	0.67
Reproducibility	0.03
Linearity	0.07
Variation with dose rate	0.19
Variation between linear accelerators	0.53
Combined standard uncertainty of well‐chamber readings	1.0

**Table 2:** Estimated relative standard uncertainty of chamber response for orthovoltage measurements

*Physical Quality or Procedure*	*Relative Standard Uncertainty (%)*	
Step 1: Absolute dose calibration of orthovoltage unit		
Long‐term stability of dosimeter	0.3	
Establishment of reference conditions	1.0	
Combined uncertainty of step 1	1.0	
Step 2: Establishment of well‐chamber reference conditions	100 kVp	250 kVp
Gantry angle uncertainty of 0.5°	0.22	0.17
FSD uncertainty of 2 mm	1.00 (0.50^a^)	1.03 (0.51^a^)
Angle of chamber relative to central axis of beam	0.07	0.07
Off‐axis position uncertainty of 2 mm	0.09	0.02
Combined uncertainty of step 2	1.03 (0.56^a^)	1.04 (0.54^a^)
Reproducibility	0.03	0.01
Linearity	1.07 (0.09^b^)	0.08
Combined standard uncertainty of well‐chamber readings	1.8 (1.2^a,b^)	1.5 (1.1^a^)

a FSD uncertainty of 1 mm.

b Dose restricted to 100−400 cGy.

When the uncertainty associated with establishing the FSD is reduced to ≤1mm and the dose restricted to the linear region of the dose‐response curve, the overall uncertainties for the measurements using 250 kVp and 100 kVp beams are reduced to 1.2% and 1.1%, respectively. If a closed‐ended applicator is used, or if the size of an open‐ended applicator is selected so that the edges of the applicator are in contact with the surface of the chamber, the requirement that the FSD error be no greater than 0.1 cm can more readily be imposed. Otherwise, each department should assess the precision of establishing the FSD for open‐ended applicators and determine the corresponding tolerance for constancy checks of the well‐type chamber.

Absolute dose calibration of the external beam units is performed monthly using an appropriate ionization chamber. The relative standard uncertainty associated with the long‐term stability of the dosimeter is 0.3%, and the uncertainties associated with establishment of reference conditions for measurements in a water phantom on a linear accelerator and for medium energy X‐ray beam are 0.4% and 1.0%, respectively.^(5)^ The combined relative standard uncertainties associated with measuring the constancy of the beam output under reference conditions for a linear accelerator and for an orthovoltage unit are 0.5% and 1.0%, respectively. If FSD errors for the well‐type chamber measurements can be reduced to no greater than 2 mm for linear accelerators or 1 mm for an orthovoltage unit, the combined relative standard uncertainty for constancy checks of the well‐type chamber response, corrected for beam output as measured with an appropriate dosimeter, can be less than or equal to 1.0% for checks performed with a linear accelerator, and less than or equal to 1.2% for checks performed with an orthovoltage unit. A tolerance of 1.2% for the constancy of the well‐type chamber can therefore be imposed. The large uncertainty associated with establishing reference conditions for output measurements of medium energy X‐rays is due to the high‐dose gradient, of up to 1% per mm, for beams at the lower end of the energy range. If this uncertainty could be reduced — for example, by measuring output in a Solid Water phantom using beams in the higher end of the energy range, and/or using the applicator with the greatest FSD — the tolerance for constancy of the well‐type chamber could be reduced. The calibration factors of the ionization chambers used to measure the dose output of the external beam units should also be regularly checked using a radioactive source with a long half‐life,^(1)^ so the use of an external beam unit to check constancy of the well‐type chamber, therefore, represents an indirect check of constancy with a source with a long half‐life.

Checking the constancy of the well‐chamber response to both megavoltage and orthovoltage X‐rays ensures that the chamber response is stable over a broad spectrum of photon energies, but checking the response using either unit alone would provide a check of the chamber stability. However, it should be noted that the dose rates of external beam units are orders of magnitude higher than those for low‐dose‐rate brachytherapy, although similar to dose rates obtained using an high‐dose‐rate brachytherapy source. The radiation from the linear accelerator is delivered as a pulsed rather than continuous beam. Reducing the dose rate of the linear accelerator changes the pulse repetition frequency rather than the dose (or dose rate) per pulse. A comparison of the response of the well‐type chamber to linear accelerator beams of different dose rates, therefore, does not approximate a comparison of the response of the well‐type chamber to high‐ and low‐dose rate continuous X‐ray radiation. The dose rate, or dose per pulse, to the surface of the well‐type chamber cannot feasibly be reduced to less than 2 Gy per hr by increasing the SSD or FSD, or by inserting absorbent materials between the source and the chamber. Hence, it is also essential to check the chamber and electrometer leakage as part of the quality assurance program for the well‐type chamber.

## IV. CONCLUSIONS

Provided reference conditions can be reproducibly established, the response of a well‐type chamber should remain constant to within 1.0% and 1.2% when exposed to a constant dose from a linear accelerator and an orthovoltage unit, respectively. However, the uncertainty for establishing reference conditions for output measurements for an orthovoltage unit can be reduced, which would justify a reduction of the tolerance for constancy measurements.

## Supporting information

Supplementary Material
