# Supplementary material for: Constancy checks of well‐type ionization chambers with external‐beam radiation units
Source: J Appl Clin Med Phys. 2015 Nov 8;16(6):508–14. doi: 10.1120/jacmp.v16i6.5608 (PMC5691027; doi:10.1120/jacmp.v16i6.5608)
Supplement: Supplementary file 1 — Supplementary Material [file ACM2-16-508-s001.docx]

**Constancy checks of well-type ionization chambers with external beam radiation units**

**Sara L Hackett^1^, Benjamin Davis^2^, Andrew Nixon^2^, Ruth Wyatt^2^**

**Affiliations:**

1: Department of Radiotherapy, University Medical Center Utrecht, Heidelberglaan 100, 3584 CX, Utrecht, Netherlands

S.S.Hackett@umcutrecht.nl

2: Medical Physics Department, University Hospitals Birmingham NHS Foundation Trust, Birmingham, B15 2TH, United Kingdom

Running title: Constancy checks of well-chambers with external beam units
